# Characteristics and Management of Children with COVID-19 in Turkey

**DOI:** 10.4274/balkanmedj.galenos.2020.2020.7.52

**Published:** 2020-10-23

**Authors:** Burcu Ceylan Cura Yayla, Yasemin Özsürekçi, Kübra Aykaç, Pembe Derin Oygar, Sibel Laçinel Gürlevik, Sare İlbay, Musa Gürel Kukul, Sevilay Karahan, Ali Bülent Cengiz, Mehmet Ceyhan

**Affiliations:** 1 Department of Pediatric Infectious Diseases, University of Health Science Turkey, Ankara Training and Research Hospital, Ankara, Turkey; 2 Department of Pediatric Infectious Diseases, Hacettepe University School of Medicine, Ankara, Turkey; 3 Department of Biostatistics, Hacettepe University School of Medicine, Ankara, Turkey

**Keywords:** COVID-19, children, favipiravir, refugees, Turkey

## Abstract

**Aims:**

Limited data about disease management strategies are available for pediatric patients with coronavirus disease-2019, particularly in Turkey. This study aimed to share the data on patients aged under 18 years in our country to be beneficial for understanding the disease course in children.

**Methods:**

A retrospective review of the medical records of pediatric patients aged under 18 years who were confirmed as coronavirus disease-2019 between March 11, and June 23, 2020, and were admitted to our hospitals was conducted.

**Results:**

A total of 220 pediatric patients with coronavirus disease-2019 were evaluated, of which 48.2% were boys, with a median age of 10 years, and 9.5% had underlying diseases. Patients were classified according to severity, with the percentages of asymptomatic, mild, moderate, and critical/severe cases determined to be 25.5%, 45%, 26.8%, and 2.7%, respectively. Extracorporeal membrane oxygenation was required in two patients (0.9%) and mechanical ventilation in three (1.4%). Targeted therapies were used in six patients (2.7%), with hydroxychloroquine being the most commonly used agent either alone (one patient) or in combination with favipiravir (five patients). Two patients (0.9%) died, and nine (4.1%) were still hospitalized during the study period.

**Conclusion:**

Although the disease course of coronavirus disease-2019 seems to be mild in children, critical illness is significant, and the treatment strategy primarily should consist of supportive care according to our preliminary observations.

A new underlying cause of pneumonia, first identified in the Wuhan region of China and began to appear in December 2019, has been identified as a new member of the coronavirus family (1). The virus, which has been named the 2019-new coronavirus (2019-nCoV) by the World Health Organization, causes a disease named coronavirus disease-2019 (COVID-19) and has spread across the world including Turkey, and it has become a serious public health problem (1,2). The data regarding COVID-19 in children continue to be characterized by uncertainties regarding epidemiological features and transmission routes, and availability has been limited. As of February 11, 2020, the China Centers for Disease Control and Prevention (CDC) has reported that only 2% of the 72,314 known cases occurred in individuals younger than 19 years (3). A recent CDC report, published April 10, 2020, revealed that 1.7% of 149,082 cases of COVID-19 infection in the United State of America were children (4). These data suggest that, although various comorbid conditions and underlying diseases have been identified among children, unlike other viruses, 2019-nCoV causes less severe disease and progresses better in children (5). Studies reporting this disease among children have indicated that disease progression is more severe and complicated in children under 2 years relative to older children; however, one case series reported that none required mechanical ventilation in nine children under the age of 1 year, and no serious complications were reported (6). In a study from China, which included 2143 cases in children, only one 14-year-old child died. In this study, 94.1% of the children being studied, with a median age of 7 years, were reported either to be asymptomatic or to follow a moderate clinical course. However, the authors of this study did not discuss the reasons why the numbers of severe and critical pediatric cases are threefold lower (5.9%) than in adults (7). In contrast, another study, which included 171 confirmed pediatric COVID-19 cases over a period of approximately 1 month, revealed the occurrence of pneumonia in 64.9% of children, and cough/pharyngeal edema were reported as the most common complaints, with fever as the third (8). In another study that examined the clinical, laboratory, and radiological features of pediatric cases, consolidation, with procalcitonin elevation and a halo sign on chest computed tomography (CT), was observed in children than in adults (9). These data suggested that the clinical and radiological features of children differ from those observed in adults, indicating that disease management and treatment in children require a different approach from those used in adults.

Few data are available regarding management strategies for children with COVID-19 (10,11). Host parameters, country-based dynamics, and drug availability used in patients with COVID-19 should all be considered, and the experiences of each country during this pandemic can greatly affect our fight with this emerging pathogen. Therefore, we believe that sharing the available data on patients aged under 18 years in our country will be beneficial for understanding the disease course in children.

## MATERIALS AND METHODS

**Figure 1:**
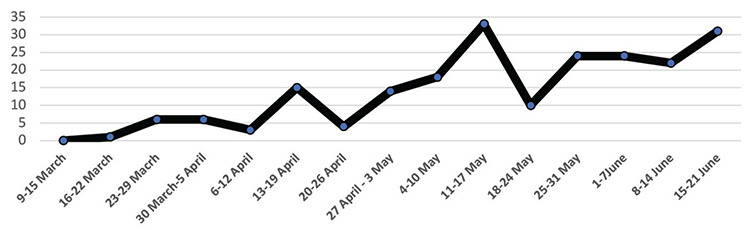
The number of patients diagnosed each week.

We conducted a retrospective review of the medical records of pediatric patients aged under 18 years, who were confirmed as COVID-19 between March 11 and June 23, 2020 (Figure 1), and were admitted to the University of Health Sciences, Ankara Training and Research Hospital and Hacettepe University School of Medicine. The study was approved by the Public Health Agency, Turkey Ministry of Health and the Ankara Educating and Training Hospital Ethical Committe, Ankara, Turkey (E-277).

We diagnosed suspected cases, according to our national COVID-19 guidelines. Criteria were changed intermittently according to changes made by the Coronavirus Scientific Advisory Board in Turkey in response to new data regarding the disease. Suspected cases with positive real-time reverse transcriptase-polymerase chain reaction or serum-specific antibodies against 2019-nCoV were accepted as confirmed cases (12).

Data regarding the demographic and clinical characteristics of patients, including age, sex, signs and symptoms, exposure history, preexisting comorbidities (i.e., heart disease, chronic lung disease, developmental delay, hematological disease, and tracheostomy), laboratory findings, chest CT and X-ray results, complications, treatments especially targeted pharmacotherapies [i.e., hydroxychloroquine, favipiravir, intravenous immunoglobulin (IVIG), tocilizumab, and convalescent plasma], respiratory support or additional organ support need [such as extracorporeal membrane oxygenation (ECMO)], and clinical outcomes were obtained from the medical records of three hospitals. The date of admission, days from illness onset to diagnosis confirmation, and history of a family cluster were recorded. Clinical outcomes at the time of data closure included survival rates for all cases with complete data.

The severity of pediatric COVID-19 cases was categorized based on the clinical characteristics, laboratory examination, and radiologic imaging results, as defined by Dong et al. (7), as follows: (a) asymptomatic infection included cases with positive diagnoses but without any clinical or radiological findings; (b) mild disease included cases with acute upper respiratory tract infections but without clinical and radiological pneumonia; (c) moderate disease included cases with pneumonia and symptoms of respiratory tract infection; (d) severe disease included cases with progressive respiratory disease, dyspnea, and central cyanosis; and (e) critically ill included cases presented with acute respiratory distress syndrome or respiratory failure, shock, and organ dysfunction, including encephalopathy, myocardial injury, coagulation abnormalities, and acute kidney injury.

### Statistical analysis

Data were evaluated using SPSS version 23.0 (SPSS, Inc., Chicago, IL, USA). Descriptive statistics were used to summarize the primary characteristics of patients, including the median and minimum-maximum for continuous variables. Frequency distribution was used for categorical variables. Chi-squared or Fisher’s Exact tests were performed to compare the categorical variables. The Kruskal-Wallis test was preferred for multiple comparisons. In all analyzes, all tests were two-tailed, and p<0.05 was considered significant.

## RESULTS

**Figure 2:**
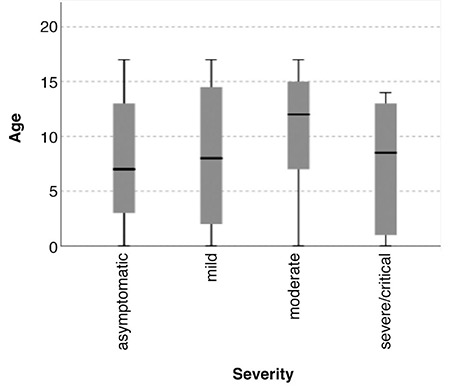
Comparisons of severity and age with Kruskal-Wallis test. The age of children with moderate clinical presentation was higher than those with mild and asymptomatic presentation (p<0.05).

Of 2,530 children who were assessed and tested, 220 (8.6%) were confirmed as COVID-19 positive. Of all, the diagnosis of COVID-19 was confirmed in 211 (96%) by reverse transcriptase-polymerase chain reaction from nasopharyngeal swabs and in 9 (4%) using the detection of serum-specific immunoglobulin (Ig) M and IgG antibodies. Of the 220 children with COVID-19, 106 (48.2%) were boys, and the median (minimum-maximum) age was 10 (0-17) years (Figure 2). No significant difference was observed in the numbers of boys and girls. Asymptomatic and mild cases accounted for 55 (25.5%) and 90 (45%) patients, respectively, representing 70.5% of all cases. The proportions of moderate and severe/critical cases were 26.8% and 2.7%, respectively. The age of children with moderate clinical presentation was higher than those with mild and asymptomatic presentation (p<0.05). We did not detect critical/severe disease course in patients older than 15 years.

Patients were hospitalized and admitted to either inpatient wards or pediatric intensive care unit (PICU), according to disease course. Only three (1.4%) patients were admitted to the PICU. The length of hospital stay was median of 10 (min-max: 10-41) days in PICU. Demographic and clinical characteristics, according to disease severity, are summarized in Table 1. Of the 220 total cases analyzed, 217 (98.6%) were family-clustered or reported a close contact history with confirmed COVID-19 patients. The transmission mechanism remained unclear in three patients.

**Figure 3:**
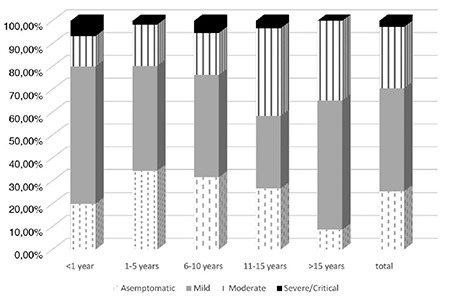
Clinical severity according to age. No patient were detected with critical/severe disease course among children older than 15 years.

Fever was present in 40.5% of cases at any time during the illness. The second most common symptom was cough (5.9% of cases), followed by fatigue/myalgia (16.4%), sore throat (16.8%), diarrhea (7.7%), headache (9.1%), vomiting (4.1%), tachypnea/dyspnea (4.1%), anosmia or ageusia (5%), and conjunctivitis (1.4%). Myalgia/fatigue was common in girls than in boys (p=0.02). The other symptoms were not seen with a different frequency between boys and girls. We analyzed patients in five groups: <1, 1-5, 6-10, 11-15, and >15 years old (Figure 3). Sore throat and myalgia/fatigue were common in patients older than 11 years (p=0.02 and p=0.001, respectively) and anosmia/ageusia and headache in patients older than 15 years (p=0.001 and p=0.01, respectively). Other symptoms were seen with similar frequency among age groups.

**Figure 4:**
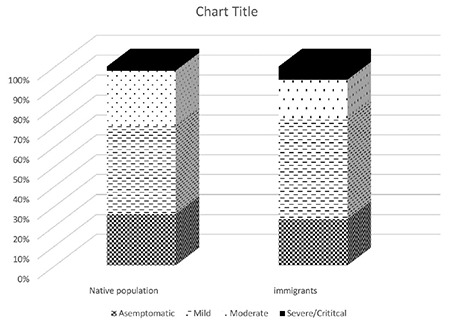
Clinical severity in native population and refugees. The clinical severity was same in both native population and refugees.

The median duration from illness onset to disease confirmation was 2 days, ranging from 0 to 20 days. All children were reported to be living in Ankara during the COVID-19 pandemic because strict isolation controls were applied to large cities, such as Ankara, after the first confirmed Turkish COVID-19 positive case on March 11, 2020. However, 30 of these patients (13.6%) were refugees from Syria or Iraq, and the clinical severity was the same in both native population and refugees (Figure 4). A total of 74 children (33.6%) presented remarkable abnormalities on X-ray or chest CT. There were no statistically significant differences in terms of lymphopenia and abnormal X-ray and chest CT (p>0.05).

Lymphopenia (13.5%) was the most common abnormal parameter in the complete blood count, followed by neutropenia (8.4%), leukopenia (6%), leukocytosis (0.5%), and thrombocytopenia (0.9%). Increased levels of C-reactive protein (CRP), procalcitonin, lactate dehydrogenase (LDH), and D-dimer were identified in 17.8%, 2.3%, 27.8%, and 18.3% of patients, respectively. Only 8/114 (6.3%) patients had high troponin levels.

Some underlying diseases were identified among this pediatric cohort: four patients had neurologic disease, four had chronic pulmonary disease, three had metabolic disease, four had endocrine disease, two had malignancy, one had rheumatologic disease, one had cardiovascular diseases, one had a hematologic disease, and one had a gastrointestinal disease.

There was no statistically significant difference in terms of gender, family history, underlying diseases, neutropenia, lymphopenia, and CRP, procalcitonin, and LDH levels between asymptomatic and symptomatic children (mild, moderate, severe, and critical; p>0.05).

Antimicrobial treatments were used in 40 (18.2%) patients because of suspicion/confirmation of bacterial superinfection. Three patients were complicated with myocardial injury, two myocarditis, one fulminant myocarditis, and one hypoxic spell. Out of 220 patients, only 3 (1.4%) required respiratory support. The first patient who had fulminant myocarditis required mechanical ventilation and ECMO. The second patient with Stevens-Johnson syndrome had ECMO support. The third patient with osteopetrosis and tracheostomy at his or her basal condition required mechanical ventilation. Continuous renal replacement therapy was not required in our pediatric cohort.

### Targeted therapies

We used various therapies to target the viral infection in six patients (2.7%) with a diagnosis of myocarditis in two, fulminant myocarditis in one, and pneumonia in three. The most commonly used agent was hydroxychloroquine, as either a single agent or in combination. Favipiravir was used in five patients in combination with hydroxychloroquine in all. IVIG was used in five children, including in combination with hydroxychloroquine and favipiravir in all and in combination with tocilizumab and convalescent plasma in one. No adverse drug reaction was detected in patients.

### Outcomes

A total of three (1.4%) patients were admitted to PICU, three required invasive ventilation, two died, and one discontinued mechanical ventilation and discharged with his or her basal need of oxygen support via tracheostomy. Both critically ill children complicated with myocarditis were discharged. The overall case fatality rate in this study was 0.9%. The patients who died were aged 1 and 7 years; both had no preexisting comorbidities and had favipiravir plus hydroxychloroquine treatment. Fulminant fatal myocarditis occurred in one of them and the other had Stevens-Johnson syndrome before exposure to 2019-nCoV. Additionally, coronary dilatation was observed in the latter one approximately 2½ weeks later after the diagnosis. Nine children (4.1%) were still hospitalized in inpatient wards. The Public Health Department was notified, and all patients were instructed to quarantine at home for 14 days after discharge.

## DISCUSSION

To the best of our knowledge, this study is one of the very few that focused on the epidemiological, clinical, and management strategies of children with COVID-19 in Turkey. Among children evaluated for suspicion of COVID-19, 8.6% of them were confirmed to have COVID-19. This rate is high because of a Turkish national pandemic policy that involves halting the chain of infections by tracking down infected patients and whomever they contact. Thus, we evaluated children with contact history or were determined to be COVID-19 positive before coming to the hospital. Of the 220 pediatric patients evaluated, 98.6% had an exposure history with an infected family member. Among our cohort, 25.5% were asymptomatic, and almost all had a history of exposure to a family member or friend with COVID-19. The detection of asymptomatic children, particularly those without clear epidemiological information, can lead to a continuous disease transmission process within the community and are significant for understanding the actual disease course. Most of our cases had mild or moderate disease courses, similar to the findings reported by other studies (7,13).

Of the 220 pediatric patients in this study, two cases needed ECMO, three with a critical disease course required invasive ventilation, and all but two who were previously healthy and needed ECMO survived. At the first few weeks of the pandemic, we were puzzled by the low frequency of reports on the management of pediatric cases with critical illness. Then, studies confirmed that severe illness in children is far less frequent than in adults (3,4). Although the disease course of children is milder than adults, when in the need of ECMO, the outcome may be worse than as expected according to the findings of this study. The disease process of all age groups should be carefully evaluated, particularly those who have subtle symptoms regardless of the underlying disease condition. In one of the largest pediatric series published to date, only one child died among 2143 children, and in another study from the US/Canada, the PICU mortality rate was 4.2% (7,11). In the most recent CDC Morbidity and Mortality Weekly Report, published on April 10, 2020, 15 children were admitted to the PICU, and three deaths were reported among 2572 pediatric cases in the US (4). In our cohort, three (1.4%) were admitted to the PICU, and two died of the 220 patients. This rate appears to be high compared with reports from other countries; however, the true death rate in Turkey must be clarified by performing additional studies that include larger populations.

We observed that the rate of girls affected by the COVID-19 outbreak is similar to boys, which is different from other epidemiological studies (7,8). Although most of the boys were asymptomatic or mild, the children who died were boys. However, the reasons why men are affected more severely than women remain unknown. In our study, the median time from symptom onset to the first hospital admission was 2 days, which was lower than that in the pediatric study of Dong et al. (7) and lower than most adult studies (14,15). The earlier hospital admission may be attributed to our national policy. This study also revealed that a considerable proportion of our patients (13.3%) were refugees, who are at a potentially increased risk of contracting contagious diseases, such as COVID-19, because of the increased numbers of family members and crowded and poor living conditions. The inclusion of refugees in the national public health system, without requiring payments or imposing legal consequence, is of crucial importance during this pandemic from a humanizing perspective; therefore, they are provided by healthcare opportunities from the Turkish Government, as a right that a person should have.

Limited data are presently available regarding the radiologic features of children with COVID-19. Compared with adults, pediatric patients with COVID-19 tend to have fewer abnormal CT findings (16). Pulmonary involvement was detected using chest X-ray in one-third of the children in this study, and chest CT was performed in 32 of 52 children because of their abnormal chest X-ray or persistent cough or fever. Chest CT was abnormal in only three patients who had normal X-ray imaging. In the literature, chest X-ray is not the recommended first choice of method because of the possibility of missed diagnosis caused by normal imaging results during the early stage of the disease (10). However, the risk of radiation exposure is a great concern when performing CT scanning in children (17). Further specific radiologic studies for both diagnosis and follow-up in children with COVID-19 seem necessary. Based on our preliminary experience, the use of chest X-ray or CT in asymptomatic children is not recommended as all asymptomatic children had normal chest X-ray or CT findings.

Viral infections can cause hematological abnormalities, including neutropenia, thrombocytopenia, and anemia (18). Ma et al. (19) reported that thrombocytopenia was found in 14% of children with COVID-19; however, only two of our patients had thrombocytopenia, and one of them had hemophagocytic syndrome caused by COVID-19 as well. Lymphopenia has been reported to occur in patients with COVID-19 at early stages and may serve as an early warning indicator for severe and critical disease (10). Consistently, lymphopenia was seen especially in critical cases and in patients with a moderate disease course in our study. This finding leads us to consider lymphopenia as a predictor of severe disease course in children.

The expert consensus statement for children with COVID-19 has indicated that most patients display increased CRP and LDH levels but normal procalcitonin levels (10). Normal CRP, procalcitonin, and LDH levels were detected in most of the children in our study, but these parameters increased in most critical patients. Consistent with adult data (20,21), LDH and CRP appear to be positively correlated with COVID-19 severity in our study. Because D-dimer has been reported to be elevated in children with severe disease course (22), we evaluated our patients in terms of D-dimer levels. We detected high D-dimer levels even among asymptomatic pediatric patients. Some expert groups have recommended anticoagulant therapy, due to the risk of disseminated intravascular coagulation and venous thromboembolism among adults (23,24), and D-dimer levels are used as an indicator of anticoagulant therapy. However, to date, no specific recommendation regarding anticoagulation therapy has been established for pediatric patients with COVID-19. Therefore, we did not use D-dimer levels as a guide to initiate anticoagulant therapy in children, and we primarily focused on the clinical status of the cases rather than the laboratory parameters.

A huge knowledge gap on the treatment of children with COVID-19 is still present because of a lack of clear evidence regarding the safety and efficiency of targeted therapies. Some experts in China do not recommend lopinavir/ritonavir, ribavirin, or chloroquine phosphate in children (10), whereas lopinavir/ritonavir and arbidol were used in another pediatric case series in China (25). Remdesivir and hydroxychloroquine, both with and without azithromycin, tocilizumab, and convalescent plasma, were used as therapeutic options in children with severe disease course in a North American study (11). In this case series, none of the patients in the asymptomatic group were treated; however, only one of the patients with a moderate disease course and all who had severe/critically ill diseases were treated with a targeted therapy. Our critically ill patients were treated with hydroxychloroquine plus favipiravir and IVIG. All the remaining patients received symptomatic treatment without targeted therapy. Additionally, the use of these drugs as therapeutic options was guided primarily by their availability. However, we did not prefer to use lopinavir/ritonavir, which was recently evaluated in a randomized controlled trial in patients with COVID-19 and was reported to have no benefits beyond standard care (26). There are still many controversial data and retracted papers about the use of hydroxychloroquine, particularly with rare data on children (27,28). These data should be considered when selecting therapeutic options as well. A couple of pediatric patients treated with the mentioned targeted therapies in this study had progressing CT findings despite the clinical improvement; as a result, we stopped using targeted therapies even for severe cases after our accumulated experiences. We believe that supportive care of pediatric cases is still the main therapeutic option as in the literature.

The disease course of COVID-19 appears to be milder in children, and the treatment was primarily based on supportive care. However, several concerns remain regarding the management of pediatric cases compared with that of adults. Increased data associated with the disease course in children and the outcomes of patients under targeted therapies will guide us in the accurate management of cases in near future.

## Figures and Tables

**Table 1:**
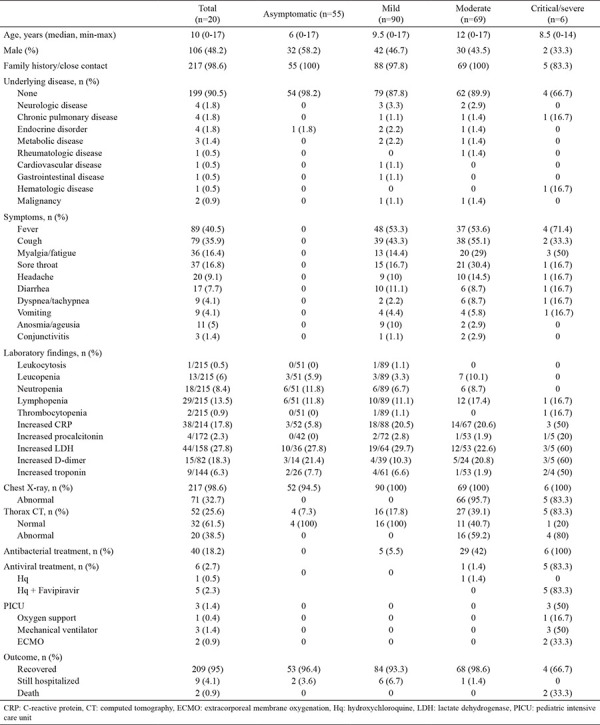
Demographic and clinical data of patients with COVID-19

## References

[1] Coronavirus disease (COVID-2019) situation reports 2020. Available from.

[2] Li Q, Guan X, Wu P, Wang X, Zhou L, Tong Y, et al (2020). Early Transmission Dynamics in Wuhan, China, of Novel Coronavirus-Infected Pneumonia. New Engl J Med.

[3] Wu Z, McGoogan JM (2020). Characteristics of and important lessons from the coronavirus disease 2019 (COVID-19) outbreak in China: summary of a report of 72314 cases from the Chinese center for disease control and prevention. JAMA.

[4] CDC COVID-19 Response Team (2020). Coronavirus disease 2019 in children-United States, February 12-April 2, 2020. MMWR.

[5] Zimmermann P, Curtis N (2020). Coronavirus Infections in Children Including COVID-19. An Overview of the Epidemiology, Clinical Features, Diagnosis, Tretament and Prevention Options in Children. Pediatr Infect Dis J.

[6] Wei M, Yuan J, Liu Y, Fu T, Yu X, Zhang Z (2020). Novel coronavirus infection in hospitalized infants under 1 year of age in China. JAMA.

[7] Dong Y, Mo X, Hu Y, Qi X, Jiang F, Jiang Z, et al. Epidemiological Characteristics of 2143 Pediatric Patients With 2019 Coronavirus Disease in China. Pediatrics doi: 10.1542/peds.2020-0702..

[8] Lu X, Zhang L, Du H, Zhang J, Li YY, Qu J, et al (2020). SARS-CoV-2 Infection in Children. New Engl J Med.

[9] Xia W, Shao J, Guo Y, Peng X, Li Z, Hu D. Clinical and CT features in pediatric patients with COVID-19 infecton: Different points from adults. Pediatr Pulmonol doi: 10.1002/ppul.24718..

[10] Shen KL, Yang YH, Jiang RM, Wang TY, Zhao DC, Jiang Y, et al. Updated diagnosis, treatment and prevention of COVID-19 in children: experts’ consensus statement (condensed version of the second edition). World J Pediatr doi: 10.1007/s12519-020- 00362-4..

[11] Shekerdemian LS, Mahmood NR, Wolfe KK, Riggs BJ, Ross CE, McKiernan CA, et al. Characteristics and Outcomes of Children with Coronavirus Disease 2019 (COVID-19) Infection Admitted to US and Canadian Pediatric Intensive Care Units. JAMA Pediatr doi: 10.1001/jamapediatrics.2020.1948..

[12] The Coronavirus Scientific Advisory Board (Turkey). 14 April 2020.

[13] Wang XF, Yuan J, Zheng YJ, Chen J, Bao YM, Wang YR, et al. Clinical and epidemiological characteristics of 34 children with 2019 novel coronavirus infection in Shenzhen. Zhonghua Er Ke Za Zhi 2020;58:E008. doi: 10.3760/cma.j.issn.0578-1310.2020.0008 [Epub ahead of print]..

[14] Huang C, Wang Y, Li X, Ren L, Zhao J, Hu Y, et al (2020). Clinical features of patients infected with 2019 novel coronavirus in Wuhan, China. Lancet.

[15] Du W, Yu J, Wang H, Zhang X, Zhang S, Li Q, et al. Clinical characteristics of COVID‑19 in children compared with adults in Shandong Province, China. Infection doi: 10.1007/s15010-020-01427-2..

[16] Chen A, Huang J, Liao Y, Liu Z, Chen D, Yang C, et al. Differences in Clinical and Imaging Presentation of Pediatric Patients with COVID-19 in Comparison with Adults. Radiol Cardiothor Imaging doi: 10.1148/ryct.2020200117..

[17] Brenner DJ, Hall EJ (2007). Computed tomography--an increasing source of radiation exposure. N Engl J Med.

[18] Pascutti MF, Erkelens MN, Nolte MA (2016). Impact of Viral Infections on Hematopoiesis: From Beneficial to Detrimental Effects on Bone Marrow Output. Front Immunol.

[19] Ma H, Hu J, Tian J, Zhou X, Li H, Laws MT, et al (2020). A Single-Center, Retrospective Study of COVID-19 Features in Children: A Descriptive Investigation. BMC Med.

[20] Chen W, Zheng KI, Liu S, Yan Z, Xu C, Qiao Z (2020). Plasma CRP level is positively associated with the severity of COVID-19. Ann Clin Microbiol Antimicrob.

[21] Zhang JJY, Lee KS, Ang LW, Leo YS, Young BE. Risk Factors of Severe Disease and Efficacy of Treatment in Patients Infected with COVID-19: A Systematic Review, Meta-Analysis and Meta-Regression Analysis. Clin Infect Dis doi: 10.1093/cid/ ciaa576 [Epub ahead of print]..

[22] Wang Y, Zhu F, Wang C, Wu J, Liu J, Chen X, et al (2020). Children Hospitalized With Severe COVID-19 in Wuhan. Pediatr Infect Dis J.

[23] Tang N, Bai H, Chen X, Gong J, Li D, Sun Z (2020). Anticoagulant treatment is associated with decreased mortality in severe coronavirus disease 2019 patients with coagulopathy. J Thromb Haemost.

[24] Kollias A, Kyriakoulis KG, Dimakakos E, Poulakou G, Stergiou GS, Syrigos K. Thromboembolic risk and anticoagulant therapy in COVID-19patients: emerging evidence and call for action. Br J Haematol doi: 10.1111/bjh.16727 [Epub ahead of print]..

[25] Song W, Li J, Zou N, Guan W, Pan J, Xu W (2020). Clinical features of pediatric patients with coronavirus disease (COVID-19). J Clin Virol.

[26] Cao B, Wang Y, Wen D, Liu W, Wang J, Fan G, et al (2020). A Trial of Lopinavir-Ritonavir in Adults Hospitalized with Severe Covid-19. N Engl J Med.

[27] Mehra M, Desai SS, Ruschitzka F, Patel AN. Hydroxychloroquine or chloroquine with or without a macrolide for treatment of COVID-19: a multinational registry analysis. Lancet doi: 10.1016/S0140-6736(20)31324-6..

[28] Mehra MR, Desai SS, Kuy S, Henry TD, Patel AN (2020). Cardiovascular Disease, Drug Therapy, and Mortality in Covid-19. N Engl J Med.

